# Factors associated with family caregiver burden of patients with dementia

**DOI:** 10.1590/1980-5764-DN-2024-0190

**Published:** 2025-06-02

**Authors:** Andressa Hermes-Pereira, Mariana Goulart, Renata Kochhann, Artur Francisco Schumacher-Schuh

**Affiliations:** 1 Universidade Federal do Rio Grande do Sul, Rio Grande do Sul RS, Brazil.; 2 Pontifícia Universidade Católica do Rio Grande do Sul, Rio Grande do Sul RS, Brazil.; 3 Hospital Moinhos de Vento, Rio Grande do Sul RS, Brazil.; 4 Hospital de Clínicas de Porto Alegre, Rio Grande do Sul RS, Brazil.

**Keywords:** Behavioral Symptoms, Affective Symptoms, Caregiver Burden, Dementia, Sintomas Comportamentais, Sintomas Afetivos, Sobrecarga do Cuidador, Demência

## Abstract

Neuropsychiatric symptoms of dementia, also known as behavioral and psychological symptoms of dementia, are extremely common. These symptoms represent one of the main causes of burden and distress among caregivers and can lead to decline in their physical and mental health.

**Objective:**

This study aimed to identify predominant characteristics in a sample of Brazilian outpatient dementia patients and explore which symptoms exert the greatest influence on caregiver burden.

**Methods:**

This was a cross-sectional study involving 119 caregivers. Stepwise regression analysis was used, with caregiver burden as the dependent variable and depressive symptoms, caregiver distress due to neuropsychiatric symptoms, and quality of life as independent variables in the final model.

**Results:**

Regression analysis identified three different models as predictors of caregiver burden. The first model, composed of depressive symptoms, predicted 34% of caregiver burden (p≤0.000). The second model, composed of depressive symptoms and caregiver distress due to neuropsychiatric symptoms, predicted 49% (p≤0.000). The third model, composed of depressive symptoms, caregiver distress due to neuropsychiatric symptoms, and quality of life, predicted 52% (p≤0.008).

**Conclusion:**

Our findings indicate that problematic behaviors in dementia patients, depressive symptoms, and quality of life are associated with the level of caregiver burden and distress. Further research is needed to differentiate these findings among different types of dementia and to better understand how caregivers’ individual characteristics influence their own burden.

## RESUMO

Os sintomas neuropsiquiátricos da demência, também conhecidos como sintomas comportamentais e psicológicos da demência, são extremamente comuns. Esses sintomas representam uma das principais causas de sobrecarga e sofrimento nos cuidadores e podem levar ao declínio da sua saúde física e mental.

**Objetivo:**

Este estudo teve como objetivo identificar as características predominantes em uma amostra de pacientes ambulatoriais brasileiros afetados por demência e explorar quais sintomas exercem maior influência na sobrecarga dos cuidadores.

**Métodos:**

Trata-se de um estudo transversal com a inclusão de 119 cuidadores. Foi utilizada uma análise de regressão *stepwise*, e o modelo final incluiu a sobrecarga como variável dependente e sintomas depressivos, desgaste do cuidador devido aos sintomas neuropsiquiátricos e qualidade de vida como variáveis independentes.

**Resultados:**

A análise de regressão identificou três modelos diferentes como preditores de sobrecarga. O primeiro modelo, composto de sintomas depressivos, predisse 34% da sobrecarga (p≤0,000). O segundo modelo, composto de sintomas depressivos e desgaste do cuidador devido aos sintomas neuropsiquiátricos, predisse 49% (p≤0,000). O terceiro modelo, composto de sintomas depressivos, desgaste do cuidador devido aos sintomas neuropsiquiátricos e qualidade de vida, predisse 52% (p≤0,008).

**Conclusão:**

Nossos resultados mostraram que os comportamentos problemáticos dos pacientes com demência, sintomas depressivos e qualidade de vida estão relacionados ao nível de carga e angústia dos cuidadores. Mais pesquisas são necessárias para distinguir esses achados entre os diferentes tipos de demência e para entender melhor como as características individuais dos cuidadores influenciam sua própria sobrecarga.

## INTRODUCTION

The prevalence of dementia worldwide in older adults is estimated to increase from 57,4 million in 2019 to 152,8 million by 2050 due to population aging^1^. Dementia is one of the most predominant neurological illnesses occurring in the older adult population. People with dementia (PWDs) worldwide are cared for by their family caregivers, commonly by their spouses or children^2^. PWDs rely on caregiver support for activities of daily living (ADL) such as bathing/showering, getting dressed, eating, walking/climbing stairs among others, which consist of self-care tasks. Caregivers have to cope with deteriorative function and progressive dementia-related symptoms of PWD. This stage includes the decrease of instrumental ADL (IADL) related to living independently or being able to take care of oneself and family^3^.

The anticipated rise in lifespan duration, alongside the gradual functional decline in patients with dementia, presents significant challenges for both formal and informal caregivers. The term “caregiver burden” encapsulates the strain and difficulties experienced by those providing care for individuals with dementia. Caregiver burden refers to the extent to which a caregiver’s emotional, physical, social, and financial well-being is affected by their responsibilities in caring for a person with dementia^4^. This definition recognizes the wide-ranging impact of caregiving, acknowledging the various forms of stress and hardships that caregivers may endure and which are linked to the cognitive impairment, behavioral disturbances, and restricted daily activities of the patients they care for. Caregivers themselves can become “secondary patients” due to the adverse effects of providing care, which may lead to deterioration in their overall health. They face an increased risk of chronic diseases, physiological changes related to health and heightened healthcare utilization^4,5^.

In addition to cognitive impairment and impact on functionality, behavioral and psychological symptoms of dementia (BPSD), also known as neuropsychiatric symptoms of dementia (NSD), are significant central characteristics of dementia. BPSD affect up to 90% of patients diagnosed with dementia during the progression of the disease^6^. The International Psychogeriatric Association established “symptoms of disturbed perception, thought content, mood, and behavior frequently occurring in patients with dementia”^7^. BPSD include apathy, depression, anxiety, psychosis, agitation, aggression, sleep disturbances, calling out repeatedly, and other problematic behaviors such as wandering, sexually inappropriate behaviors, and care refusal^8,9^.

Previous studies have described factors associated with caregiver burden, including cognitive function, stages of dementia, depression, ADL, IADL, and BPSD. It was shown that behavioral problems or psychological symptoms were the primary factors associated with caregiver burden in people with dementia^10,11^. Given the likely increase in the number of people living with dementia, the complexity of the burden and the negative impact on the caregiver, it is essential to examine the caregiving burden among informal caregivers of persons diagnosed with dementia. This study aimed to ascertain the predominant characteristics that could predict burden in caregivers within a sample of Brazilian outpatients affected by dementia and to explore which symptoms have the greatest impact on caregiver burden.

## METHODS

### Study design and sample

A cross-sectional study was conducted between August 2017 to March 2020. The data for this study were retrieved from a previous single-blinded, parallel-group, multicenter, randomized clinical trial with a 1:1 allocation rate. That report follows the CONSORT recommendations, and the entire protocol is available in Hermes-Pereira (2021), trial registration number: NCT03260608. We included the participants who were in the research at baseline.

Briefly, female family members were included in the study if:

- The cared person had a medical dementia diagnosis (of any etiology and severity);
- The caregiver provided in-home unpaid care;
- The caregiver had responsibility over the patient for at least 40 hours a week, for a minimum of six months; and
- The caregiver had at least four years of formal education.

Exclusion criteria included:

- Physical limitations that prevent the application of research instruments;
- Caregivers of patients with significant functional impairment related to other diseases rather than dementia; and
- Planning to transfer the person with dementia to a nursing home in the following six months.

In this present study, a total of 119 female caregivers responsible for the care of older adult with dementia were included. It was decided to exclude male caregivers, as evidence suggests that gender differences significantly influence caregiving approaches and experiences. Before their involvement, all participants provided written informed consent. If any difficulties arose during the completion of the questionnaires, the researcher or a designated research assistant guided to facilitate the comprehensive addressing of all inquiries. Such protocols were implemented to adhere to ethical standards and to ensure meticulous data acquisition.

### Variables

The dependent variable in this study was caregiver burden, measured by the Zarit Burden Interview (ZBI)^12^. ZBI consists of 22 items that assess the burden experienced by caregivers on several aspects of their lives: health, personal life, social, financial circumstances, etc. A possible total score ranges from 0 to 88 with a higher score indicating an increase in caregiver burden.

The independent variables that were examined in this study included:

- The Neuropsychiatric Inventory Questionnaire (NPI-Q)^13^ was used to evaluate BPSD. This instrument consists of 12 domains that assess the severity level of neuropsychiatric symptoms of PWDs (e.g., delusions, hallucinations, dysphoria, anxiety, etc.) and the effect of the symptoms on caregiver distress;
- The Beck Depression Inventory (BDI-II)^14^ was used to detect and measure the severity of depressive symptoms; and
- The World Health Organization Quality of Life Questionnaire (WHOQOL)^15^ was used to measure caregiver’s quality of life; it is a self-assessment scale that encompasses four quality of life domains (psychological, physical health, social relations, and the environment).

### Statistical analysis

Descriptive statistics were used to summarize the sociodemographic status and clinical characteristics. Mean and SD were reported for continuous variables, and categorical variables, counts, and proportions were reported. The Spearman Correlation was used to test the association between caregiver burden, anxious symptoms, depression symptoms, quality of life, neuropsychiatric symptoms of dementia, and patient functionality. Only the statistically significant variables were then entered into the stepwise regression analysis, anxious symptoms and patient functionality were not statistically significant. The final model consisted of those variables with a p<0.05. The study results are presented as regression coefficient (β) along with its 95% confidence intervals (CIs) and p-values. The Statistical Package for the Social Sciences (SPSS) V.25.0 was used to analyze the data.

The final model consisted of burden caregiver as dependent variable, and independent variables: depressive symptoms, caregiver distress due to neuropsychiatric symptoms of dementia, and quality of life.

### Ethics approval and consent to participate

The primary data collection used in this study obtained ethics approval from the Ethics Committee of Hospital de Clínicas de Porto Alegre, Hospital São Lucas (HSL), and Hospital Nossa Senhora da Conceição (HNSC), all located in Porto Alegre, Brazil. The Ethics Committee at each hospital permitted the data collection. Before participating in this study, the participants (caregivers) were provided with information about the background and aim of the study, the benefits and risks, and the compensation they would receive for their participation. In addition, the participants were also informed about their rights to withdraw from this study at any time without any consequences. All the participating caregivers gave written informed consent. All methods were carried out in accordance with relevant guidelines and regulations.

## RESULTS

A total of 119 participants were recruited. Demographic information is presented in Table 1. The caregivers had an average age of 53.70, with a mean educational level of 11.64 years. The average caregiving time was 4.46 years. Most of the sample were daughters of dementia patients (68.1%), while 21.8% were wives; others were sisters, granddaughters, daughters-in-law, or other relatives (2.5%). Regarding marital status, nearly half of the sample were married (48.7%), 22.7% were single, and 20.2% were divorced. Most caregivers were unemployed (63.9%), and 36.1% worked part-time (Table 1).

**Table 1:** Demographic characteristics of caregivers.

Variables related to caregivers		n (%)
Age, mean (SE)		53.70 (11.31)
Education in years — mean (SE)		11.64 (4.32)
Duration of caregiving in years, mean (SE)		4.46 (3.99)
Relationship between the patient and the primary caregiver — n (%)	Spouse	26 (21.8)
	Child	81 (68.2)
	Sister	3 (2.5)
	Grandchild	3 (2.5)
	Daughter-in-law	3 (2.5)
	Other	3 (2.5)
Marital status, n (%)	Married/in couple	58 (48.7)
	Widowed	2 (1.7)
	Divorced/ separated	24 (20.2)
	Single	27 (22.7)
	Common-law marriage	8 (6.7)
Employment status, n (%)	Not working	76 (63.9)
	Working part-time	43 (36.1)

The final results of the regression model are presented in Table 2. Three variables were significant risk factors: depressive symptoms, the distress of neuropsychiatric symptoms, and quality of life. Three models were created: the first with a role and predictor of 34% of the burden (p≤0.000), while the second model composed of depressive symptoms and distress of neuropsychiatric symptoms predicted 49% of caregivers’ burden (p≤0.000). The third model predicted 52% of the burden and it was composed of depressive symptoms, distress of neuropsychiatric symptoms, and quality of life (p≤0.008).

**Table 2:** The final model in Stepwise Regression Analysis.

Dependent variable		Predictor	B	SE	*𝞫*	T	p	ΔR2	F change	Sig F change
Burden	Model 1	Depressive symptoms	0.801	0.104	0.582	7.732	<0.000	0.338	59.779	<0.000
	Model 2	Depressive symptoms	0.596	0.097	0.432	6.128	<0.000	0.495	35.969	<0.000
		Caregiver distress (NPS)	0.600	0.100	0.423	5.997	<0.000			
	Model 3	Depressive symptoms	0.492	0.102	0.357	4.807	<0.000	0.525	7.282	<0.008
		Caregiver distress (NPS)	0.548	0.099	0.386	5.519	<0.000			
		Quality of life	-3.813	1.413	-0.198	-2.698	<0.008			

## DISCUSSION

The study aimed to evaluate which symptoms exert the most significant influence on caregiver burden. We considered caregiver burden as an umbrella term consisting of different domains, such as the caregiver’s emotional, physical, social, and financial well-being^4^. Although caregivers of patients with dementia often bear a significant burden, there is a lack of research addressing this population in Brazil. Few studies have specifically investigated the challenges these caregivers face within the Brazilian context; this is one of the first studies in Brazil exploring the trajectory of caregiver burden in informal caregivers for patients with dementia. Our findings demonstrated that there are three risk factors shared by burden experience: depressive symptoms, caregiver distress from NPS, and quality of life.

Among these three risk factors, model one predicted 34% of the caregiver burden: depressive symptoms. These symptoms have been consistently reported as a relevant risk factor for caregiver burden^16^. It shows that caregiver attributes such as personality traits, coping strategies, and skills are also significant determinants and regarded as mediators between the influence of the patients’ behavioral issues and caregiver burden^17^. There are some explanations for this finding. Almost all caregivers were cohabitating with the patient with dementia, which might place a heavier patient care load on them. Hence, given that more patient care load and living arrangements with the patient are associated with caregiver burden, it is logical to expect that spouse caregivers will experience a higher burden compared to other caregivers. However, most of the sample of this study were daughters of dementia patients (68,1%), unemployed (63.9%) and had high education (11.64 years), while 21.8% were wives. Typically, in Brazil, the responsibility of caregiving falls solely on the patient’s family, as public support may not be available, and private sector support is costly and accessible to only a few. The lack of comprehensive governmental policies and structured social assistance programs exacerbates the issue, leading to physical, emotional, and financial strain on caregivers^18^.

The burden faced by caregivers of older adults with dementia exhibits gender differences, influenced by cultural, social, and economic factors. All participants of this study were female due to the significant evidence that women constitute the majority of family caregivers, often assuming this responsibility due to social and cultural expectations that associate caregiving with the female gender. In contrast, male caregivers tend to adopt more practical and task-oriented approaches to care, seeking external solutions and demonstrating greater willingness to accept help. They also receive greater social recognition for taking on the caregiving role, which may contribute to a lower perceived burden. Women frequently internalize the duty of caregiving as a natural extension of their familial role, whereas men, even when serving as caregivers, may not experience the same intensity of burden due to different social expectations^19^.

Model two predicted 49% of caregiver burden, which was influenced by depressive symptoms and care distress resulting from the NPS. Findings from this study are mainly consistent with results from previous studies on dementia, which found behavioral and neuropsychiatric symptoms over time were associated with an increase in caregiver burden^20^. Both positive (e.g., agitation, night time wandering) and negative neuropsychiatric symptoms (e.g., depression, apathy) were the highest predictors of caregiver burden. This higher number may potentially be due to considering depression as an NPS. NPS of dementia may result from the difficulty in understanding specific behaviors and the constant need to combat the negative stigma associated with such behaviors.

Dementia is traditionally believed to primarily affect the oldest-old, those over 85 years old, which may overshadow its significance in the youngest-old, those between the ages of 65 and 74 years, and the middle-old, those between the ages of 75 and 84 years. Therefore, this study emphasizes the importance of studying and understanding the specific factors that may influence caregiver burden for those caring for youngest-old and middle-old dementia patients. Managing neuropsychiatric symptoms, providing strong caregiver support, and educating caregivers is critical. Support services such as counseling and social services tailored for these younger patients and their caregivers should also be more readily available. We can contribute to improving studies by exploring effective psychoeducational approaches to alleviate caregivers’ burden, enhance caregivers’ engagement, and improve their quality of life. Future research should identify how cultural differences impact coping styles. Additionally, psychoeducational interventions for caregivers should incorporate culturally-specific positive coping techniques.

Limitations of this study include the relatively small sample size; however, the detailed clinical and caregiver burden characterization of this sample was sufficient to identify risk factors for caregiver burden. Furthermore, participants recruited for this study were Brazilians; therefore, the generalizability to non-Brazilian cohorts remains unknown. Larger collaborative studies involving subjects from diverse cultural and geographic regions will be necessary.

Future research should persist in exploring the intricate issue of caregiver burden and be directed towards assessing whether certain types of dementia impose greater challenges than others. Moreover, further studies should investigate gender differences among caregivers, with a focus on strategies to encourage and adequately prepare male for caregiving, as well as assess these implications on the burden experienced by female caregivers. Also, exploring individual characteristics can predict the burden and distress experienced by caregivers. A comprehensive understanding of the daily challenges encountered by caregivers of dementia patients is essential for devising impactful and efficient interventions.

## Data Availability

The datasets generated and/or analyzed during the current study are available from the corresponding author upon reasonable request.
